# Mitosis-specific phosphorylation of Mis18α by Aurora B kinase enhances kinetochore recruitment of polo-like kinase 1

**DOI:** 10.18632/oncotarget.22707

**Published:** 2017-11-27

**Authors:** Minkyoung Lee, Ik Soo Kim, Koog Chan Park, Jong-Seo Kim, Sung Hee Baek, Keun Il Kim

**Affiliations:** ^1^ Creative Research Initiatives Center for Chromatin Dynamics, Department of Biological Sciences, Seoul National University, Seoul 08826, South Korea; ^2^ Department of Biological Sciences, Cellular Heterogeneity Research Center, Sookmyung Women's University, Seoul 04310, South Korea; ^3^ Center for RNA Research, Institute for Basic Science, Department of Biological Sciences, Seoul National University, Seoul 08826, South Korea

**Keywords:** Mis18α, Aurora B kinase, PLK1, mitosis-specific phosphorylation, polo box domain

## Abstract

Mis18α, a component of Mis18 complex comprising of Mis18α, Mis18β, and M18BP1, is known to localize at the centromere from late telophase to early G1 phase and plays a priming role in CENP-A deposition. Although its role in CENP-A deposition is well established, the other function of Mis18α remains unknown. Here, we elucidate a new function of Mis18α that is critical for the proper progression of cell cycle independent of its role in CENP-A deposition. We find that Aurora B kinase phosphorylates Mis18α during mitosis not affecting neither centromere localization of Mis18 complex nor centromere loading of CENP-A. However, the replacement of endogenous Mis18α by phosphorylation-defective mutant causes mitotic defects including micronuclei formation, chromosome misalignment, and chromosomal bridges. Together, our data demonstrate that Aurora B kinase-mediated mitotic phosphorylation of Mis18α is a crucial event for faithful cell cycle progression through the enhanced recruitment of polo-like kinase 1 to the kinetochore.

## INTRODUCTION

Accurate segregation of duplicated chromosomes is crucial for daughter cells to have one copy of each chromosome during cell division. To complete accurate segregation, the chromosome should be condensed properly and mitotic spindle should bind to kinetochore bi-oriented [1]. The kinetochore is formed on a chromosomal locus called centromere that is composed of DNA segments and histone proteins containing centromere-specific H3 variant, CENP-A [2]. Mis18 complex (Mis18α, Mis18β and M18BP1) in higher eukaryote is a critical factor for the recruitment of newly synthesized CENP-A to the centromere at early G1 phase [3, 4].

Previously, it has been reported that deletion of *Mis18α* in mice causes embryonic lethality as well as defect in epidermal stratification, which are accompanied with CENP-A loss at the centromere and defects in chromosome segregation [3, 5]. Mis18 complex localizes to the centromere from telophase to early G1 phase of cell cycle prior to the CENP-A deposition to centromere [4, 6]. Phosphorylation of M18BP1 is involved in the regulation of the timing of centromere localization and licensing function of Mis18 complex. CDK1/2-mediated phosphorylation of M18BP1 on multiple sites blocks its interaction with Mis18α/Mis18β and hence centromere localization during S/G2/M phases, whereas phosphorylation of M18BP1 by PLK1 at early G1 phase facilitates centromere localization of Mis18 complex and its licensing function [7, 8].

Among mitotic kinases, Aurora serine/threonine kinases work crucially during mitosis. Aurora A kinase locates pericentrosome and regulates mitotic spindle assembly, centrosome separation and G2/M transition at the beginning of mitosis [9, 10]. Aurora B kinase locates innercentromere from prometaphase to metaphase regulating chromatin modification and chromatid separation, and relocates to midzone for cytokinesis [11]. Phosphorylation of Aurora B targets in the innercentromere participates in spindle checkpoint and regulates the microtubule-kinetochore interaction [12, 13]. Dephosphorylation of the Aurora B targets gives strong tension between microtubule and kinetochore allowing the cells to go to anaphase [1].

Recently, Aurora B kinase-PLK1-MCAK (mitotic centromere-associated kinesin) axis has been shown to be required for accurate chromosome segregation [14]. At the kinetochore, Aurora B kinase activates PLK1 by phosphorylation and the activated PLK1 in turn phosphorylates MCAK, which is essential for accrurate chromosome segregation with its increased microtubule depolymerase activity. Inhibition of either Aurora B kinase or PLK1 reduces MCAK phosphorylation on PLK1 target sites and induces formation of impolar mitotic spindle and the chromatin bridges. Interestingly, PLK1 is also needed for the full activation of Aurora B kinase at the beginning of prometaphase. Aurora B kinase, Survivin, INCENP, and borealin are members of chromosomal passenger complex (CPC) and Survivin phosphorylation by PLK1 elicits Aurora B kinase acitivity around kinetochore [15]. Thus, the cooperation between Aurora B kinase and PLK1 is a very important biological process for accurate chromosome segregation.

In this study, we report that Aurora B kinase phosphorylates Mis18α during mitosis, specifically at prometaphase which is critical for the faithful chromosome segregation. During prometaphase, microtubule dynamically interacts with kinetochore for the proper attachment and the process is regulated by Aurora B kinase and PLK1. Notably, we found Mis18α phosphorylation by Aurora B kinase is important for the recruitment of PLK1 to the kinetochore and for preventing the mitotic defects.

## RESULTS

### Mis18α is phosphorylated during mitosis by Aurora B kinase

**Figure 1:**
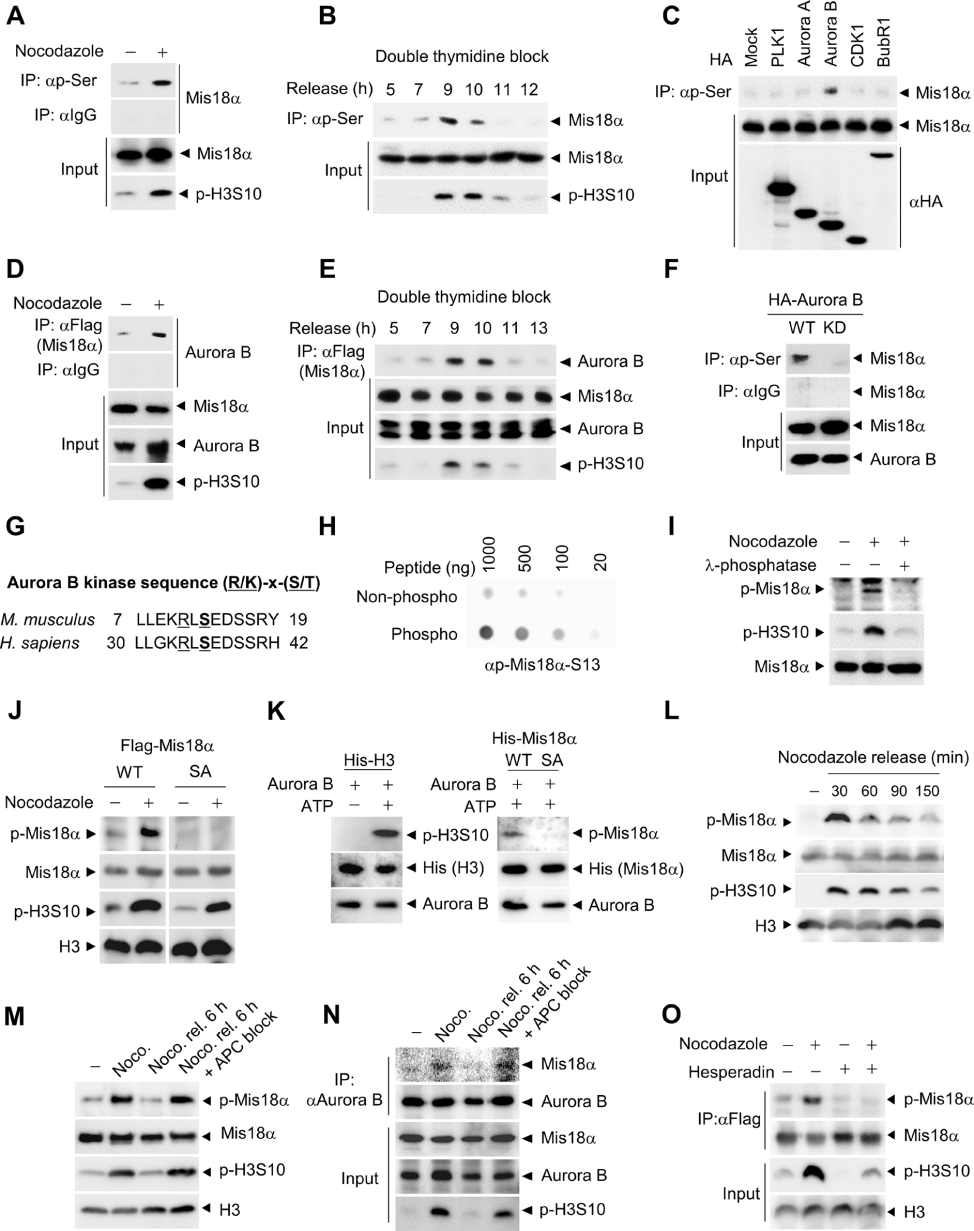
Mis18α is phosphorylated during mitosis by Aurora B kinase (**A**) HeLa cells stably expressing Flag-Mis18α (HeLa/Flag-Mis18α) were synchronized by nocodazole treatment. Cell extracts were subjected to immunoprecipitation (IP) with an antibody against phosphorylated-serine (p-Ser) followed by immunoblotting with anti-Flag antibody. Phosphorylation of 10th serine residue of histone H3 (p-H3S10) was used as a mitosis indicator. (**B**) HeLa/Flag-Mis18α cells were synchronized at G1/S by double thymidine block and released into indicated time points and were analyzed as in (A). (**C**) Mitotic kinases were transfected into HeLa/Flag-Mis18α cells and cell extracts were applied to IP with the anti-p-Ser antibody followed by immunoblotting with anti-Flag antibody. (**D**) Mitotically arrested HeLa/Flag-Mis18α cells with nocodazole treatment were applied for IP with anti-Flag antibody and detected with anti-Aurora B antibody. (**E**) HeLa/Flag-Mis18α cells prepared as in B were used for IP assay with anti-Flag antibody and detected with anti-Aurora B antibody. (**F**) HeLa/Flag-Mis18α cells transfected with Aurora B wild-type (Aurora B WT) or K160A kinase dead mutant (Aurora B KD) were used for IP with anti-p-Ser antibody. (**G**) Aurora B kinase consensus sequences in mouse and human Mis18α. (**H**) Dot blot analysis for a phosphorylation-specific antibody of Mis18α on Ser36 (p-Mis18α) by comparing non-phospho peptide with phospho-peptide at indicated concentrations. (**I**) Extracts from 293T cells transfected with Flag-Mis18α were treated with λ-phosphatase and used for immunoblotting with anti-p-Mis18α antibody. (**J**) 293T cells were transfected with Flag-Mis18α WT, Flag-Mis18αSA and synchronized by nocodazole treatment. Cell extracts were used for immunoblotting with anti-p-Mis18α antibody. (**K**) Recombinant His-H3 or His-Mis18α were incubated with purified Aurora B kinase in the presence of ATP for 30 min at 30°C for *in vitro* kinase assay. Phosphorylation of Mis18α was detected using anti-p-Mis18α. (**L**) 293T cells expressing Flag-Mis18α WT were synchronized by nocodazole treatment. After releasing, cells were harvested at indicated time points and applied for immunoblotting. (**M**) 293T cells expressing Flag-Mis18α WT were released for 6 h from nocodazole-mediated synchronization with or without MG132 treatment to block APC activity. (**N**) HeLa/Flag-Mis18α cells were released for 6 h from nocodazole-mediated synchronization as in M and subject to IP analysis with anti-Aurora B antibody. (**O**) HeLa/Flag-Mis18α cells expressing Flag-Mis18α were treated with Aurora B kinase inhibitor, Hesperadin and the phosphorylation of Mis18α was evaluated by using anti-p-Mis18α antibody under nocodazole treatment.

Although Mis18α has been shown to function as a licensing factor for the recruitment of newly synthesized CENP-A to centromere at G1 phase, whether Mis18α is involved in the processes of cell division cycle has not been investigated. As Mis18α protein level is not changed through the cell cycle, we anticipated that post-translational modification of Mis18α might act as a signal for the regulating Mis18α function. Therefore, we analyzed whether Mis18α is phosphorylated during cell cycle progression by the mitotic kinases that actively regulate mitosis. HeLa cells stably expressing Flag-Mis18α were mitotically synchronized by nocodazole treatment and the phosphorylation level of Mis18α was analyzed. Interestingly, we detected increased phosphorylation level of Mis18α from the mitotic cell extracts comparable to the H3S10 phosphorylation, a mitotic marker (Figure 1A). Consistently, Mis18α phosphorylation increased at mitotic phase after release from G1/S cell cycle synchronization by double thymidine block (Figure 1B), confirming mitosis-specific phosphorylation of Mis18α. We next screened for potential kinases that are responsible for Mis18α phosphorylation during mitosis. Among several mitotic kinases tested, only Aurora B kinase was able to phosphorylate Mis18α (Figure 1C). We also found the increased binding between Mis18α and Aurora B kinase during mitosis (Figure 1D and 1E), which matches well with the phosphorylation pattern of Mis18α. Furthermore, the kinase dead (KD) mutant of Aurora B kinase failed to phosphorylate Mis18α (Figure 1F), indicating that Aurora B kinase activity is crucial for Mis18α phosphorylation.

Next, we searched for the phosphorylation site by Aurora B kinase in Mis18α, which is recognized by the consensus sequence, [R/K]-X-[S/T], and found that Mis18α contains only one serine residue that matches with the consensus sequence in both mouse (Ser13) and human (Ser36) (Figure 1G). LC-MS/MS analysis of Mis18α from mitotically synchronized 293T cells confirmed Ser36 as the phosphorylation site during mitosis (Supplementary Figure 1). Thus, we generated phosphorylation-specific antibody against the peptide of Mis18α and the resulting antibody detected phosphorylated form of peptide much stronger than non-phospho peptide control (Figure 1H). We then evaluated the specificity of our phospho-specific Mis18α antibody. The antibody detected a specific band corresponding to Mis18α only in nocodazole-treated cell extracts and λ-phosphatase (a Ser/Thr/Tyr phosphatase) treatment abolished the signal (Figure 1I). In addition, the antibody efficiently detected the increased phosphorylation of Mis18α WT in nocodazole-arrested cells. However, the expression of Mis18α SA, a mutant Mis18α containing alanine substitution of Ser36, did not give rise to any significant signal (Figure 1J). In an attempt to clarify the time of Mis18α Ser36 phosphorylation with this new phospho-specific antibody, the antibody generated the strongest signal during mitosis in consistency with H3S10 phosphorylation (Supplementary Figure 2A). Furthermore, synchronization of cells by mitotic drugs other than nocodazole, such as monastrol and taxol, increased phosphorylation of Mis18α (Supplementary Figure 2B). In an *in vitro* kinase assay with bacterially expressed Mis18α, purified active Aurora B phosphorylated wild-type Mis18α but not Mis18α SA (Figure 1K). Mis18α phosphorylation increased in mitotic cells, but decreased as the cells exited mitosis (Figure 1L). MG132, which induces metaphase arrest by inhibiting APC-mediated proteolysis [17], maintained Mis18α phosphorylation in parallel with H3S10 phosphorylation, although the cells were released from nocodazole-mediated arrest (Figure 1M). Concurrently, the binding between Aurora B kinase and Mis18α increased during mitosis and decreased as the cells exited mitosis, but not under APC block, in parallel with Mis18α phosphorylation pattern (Figure 1N). Moreover, the treatment of cells with Aurora B kinase inhibitor, Hesperadin [16] diminished the phosphorylation of Mis18α induced by mitotic arrest (Figure 1O), indicating that Aurora B kinase is responsible for the phosphorylation of Mis18α. Taken together, these results indicate that the phosphorylation of Mis18α is a mitosis-specific event mediated by Aurora B kinase.

### Mis18α phosphorylation is necessary for faithful mitotic division

**Figure 2:**
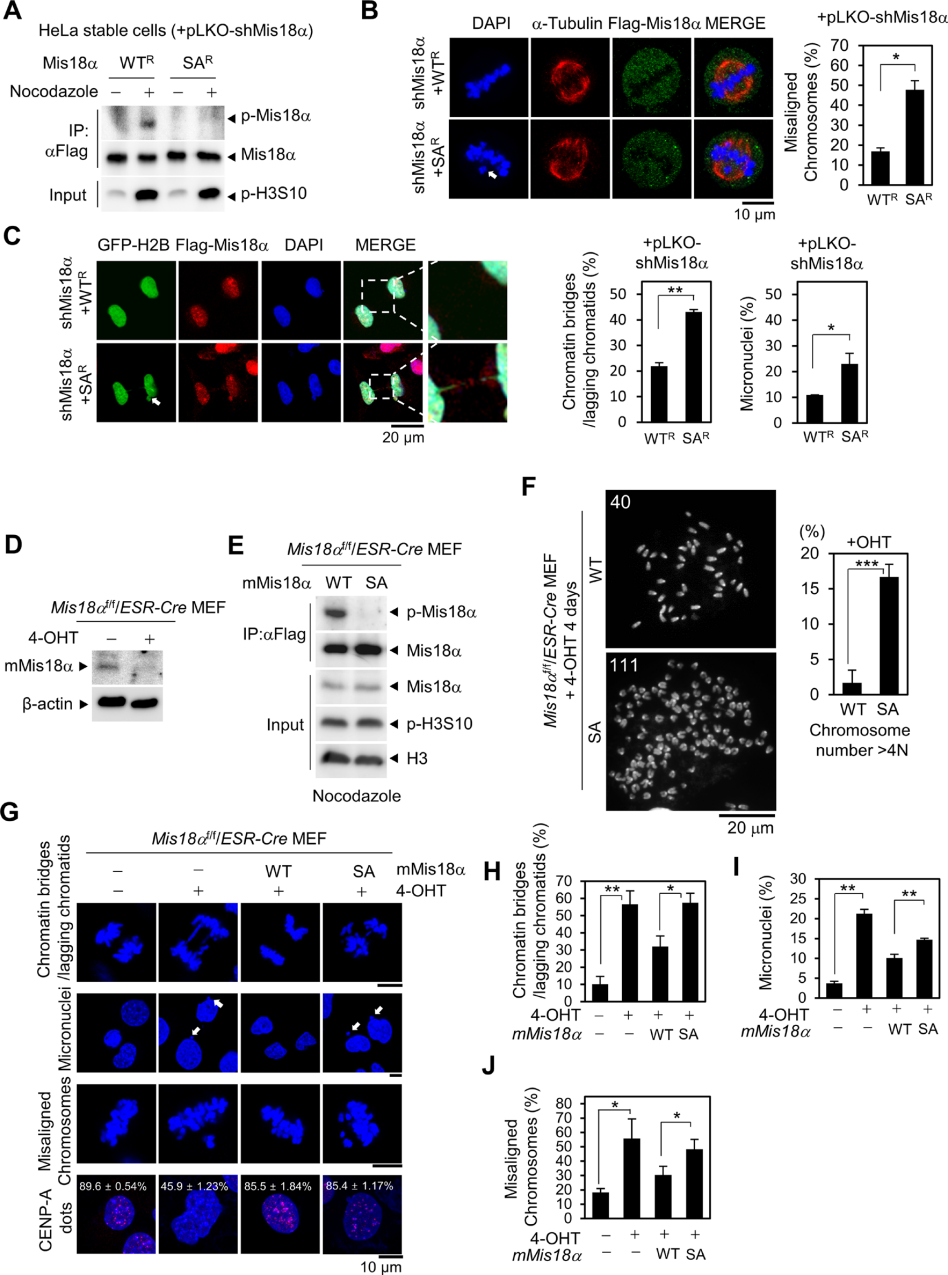
Mis18α phosphorylation is necessary for faithful mitotic division (**A**) Immunoblotting for Mis18α phosphorylation in nocodazole-synchronized HeLa/Flag-Mis18α stable cells. WT^R^ or SA^R^ represents shRNA-resistant form of Mis18α proteins. (**B**) Images of misaligned chromosomes in HeLa/Flag-Mis18α stable cells (left) and the number of cells showing misaligned chromosomes presented in percentage (right). *P* value is calculated by *t*-test (^*^p < 0.05). (**C**) Images of the chromatin bridges or lagging chromatids and micronuclei in HeLa/Flag-Mis18α stable cells (left) and the number of cells showing the chromatin bridges or lagging chromatids presented in percentage (right). *P* value is calculated by *t*-test (^*^p < 0.05). (**D**) *Mis18α^f/f^/ESR-Cre* MEFs were treated with 4-hydroxy-tamoxifen (4-OHT) for four days and the depletion of endogenous mMis18α was validated by immunoblot using anti-mMis18α antibody. (**E**) The phosphorylation of mMis18α proteins in *Mis18α^f/f^/ESR-Cre* MEFs reconstituted with Flag-mMis18α WT or Flag-mMis18α SA were validated by immunoblotting with anti-p-Mis18α antibody. (**F**) Reconstituted *Mis18α^f/f^/ESR-Cre* MEFs were analyzed by chromosome spreading assay. Histogram in right side shows the percentage of cells containing chromosome number over 4 N for each genotype. The number of chromosome spreads is 120 each and chromosomes were counted using Image J software by identifying the centromeres, the brighter part than the rest of the chromosome. *P* value is calculated by *t*-test (^***^p < 0.001). (**G**) The chromatin bridges or lagging chromatids, micronuclei and misaligned chromosomes were analyzed in reconstituted *Mis18α^f/f^/ESR-Cre* MEFs in parallel with CENP-A dots. (**H**–**J**) The percentage of cells showing the chromatin bridges or lagging chromatids (H), micronuclei (I), and misaligned chromosomes (J) was calculated from the images in G. *P* value is calculated by *t*-test (^*^p < 0.05, ^**^p < 0.01).

To find out the role of Mis18α phosphorylation, we generated HeLa cells stably expressing shRNA-resistant form of Mis18α WT (WT^R^) or Mis18α SA (SA^R^). The knockdown of endogenous Mis18α was achieved by lentiviral infection of pLKO-shMis18α just before experiment. The infection of cells with lentivirus reduced the level of endogenous Mis18α efficiently (Supplementary Figure 3). The specific phosphorylation of reconstituted Mis18α proteins was validated by immunoblot analysis (Figure 2A). With these reconstituted cell lines, we then checked the cell division and interestingly, the number of cells showing misaligned chromosomes (white arrow) at metaphase increased 2.5-fold in Mis18α SA-reconstituted cells compared with Mis18α WT-reconstituted cells (Figure 2B). In addition, either the chromatin bridges or lagging chromatids and micronuclei (white arrow) increased approximately two folds in Mis18α SA-reconstituted cells than Mis18α WT-reconstituted cells (Figure 2C).

To verify whether Mis18α phosphorylation is necessary for mitosis, we next examined the mitotic defects in *Mis18α^f/f^/ESR-Cre* MEFs in which endogenous Mis18α can be depleted by the treatment with tamoxifen (Figure 2D). *Mis18α^f/f^/ESR-Cre* MEFs were reconstituted with either Mis18α WT or Mis18α SA, and we detected mitotic phosphorylation of Mis18α only in Mis18α WT-reconstituted MEFs, but not in Mis18α SA-reconstituted MEFs (Figure 2E). Interestingly, the depletion of endogenous Mis18α induced an increase of aneuploidy in Mis18α SA-reconstituted MEFs compared with Mis18α WT-reconstituted MEFs; especially the population of cells containing the number of chromosome over 4N were increased in Mis18α SA-reconstituted MEFs compared with Mis18α WT-reconstituted MEFs (Figure 2F and Supplementary Figure 4). This is consistent with the previous studies that showed Aurora B depletion or overexpression increasing aneuploidy [18, 19]. Furthermore, the depletion of endogenous Mis18α increased the number of the chromatin bridges or lagging chromatids by four times when compared with untreated control cells (Figure 2G). While Mis18α WT-reconstitution significantly reduced the chromatin bridges or lagging chromatids formation in *Mis18α*-deficient MEFs, Mis18α SA-reconstitution failed to do so (Figure 2G and 2H). In addition, the micronuclei formation and chromosome misalignment also increased in *Mis18α*-deficient MEFs and these were not recovered by Mis18α SA-reconstitution similarly to the chromatin bridges or lagging chromatids formation (Figure 2G, 2I and 2J). However, CENP-A dots were intact indicating that these mitotic defects in Mis18α SA-reconstituted MEFs are independent of CENP-A deposition. Taken together, mitotic phosphorylation of Mis18α by Aurora B kinase is necessary both for the faithful segregation of chromosome.

### Mis18α phosphorylation is not required for CENP-A loading

**Figure 3:**
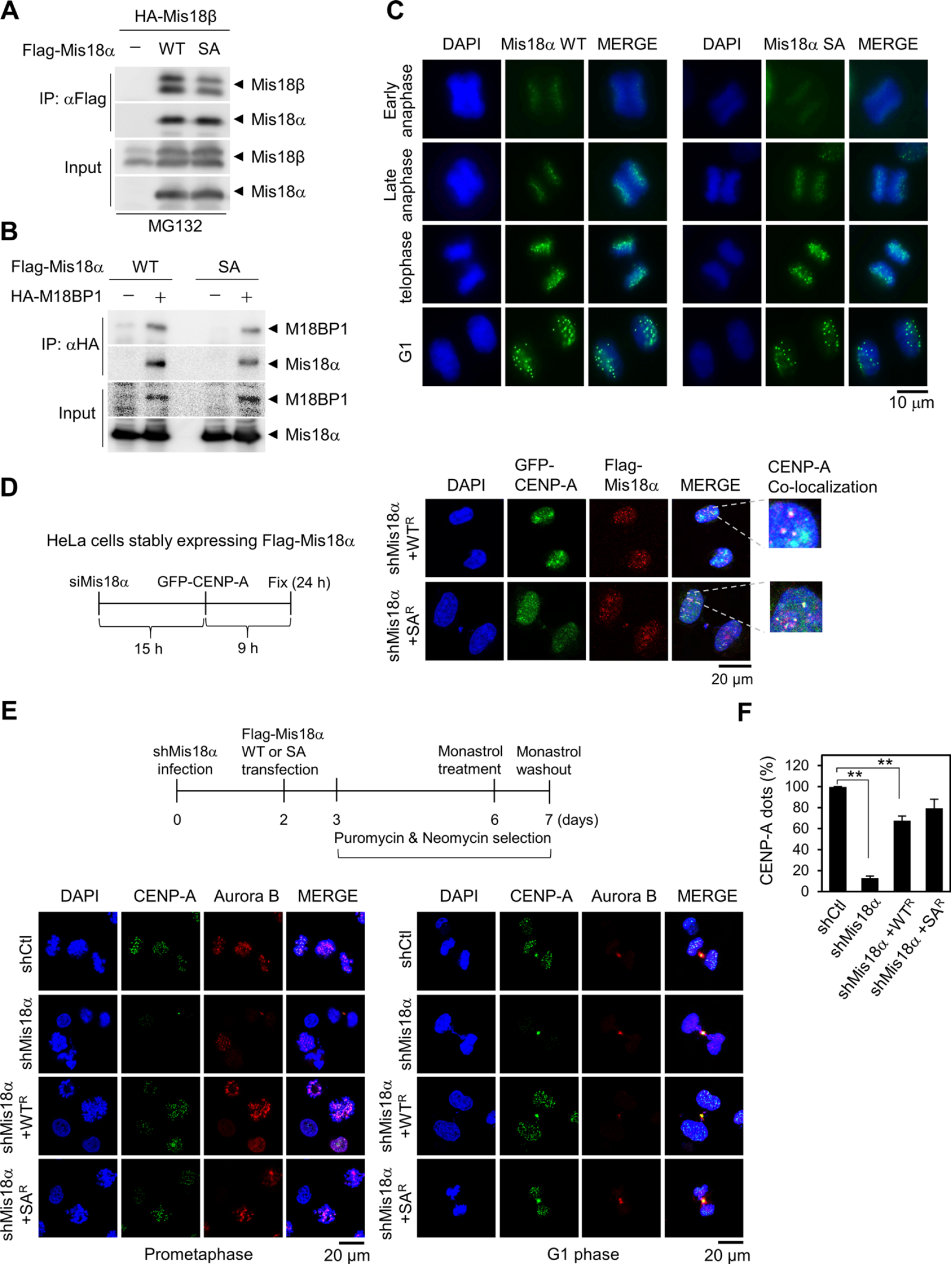
Mis18α phosphorylation is not required for CENP-A loading (**A**) HA-Mis18β with either Flag-Mis18α WT or Flag-Mis18α SA were transfected into 293T cells and the extracts were applied for co-IP assay. (**B**) The binding between Flag-Mis18α and HA-M18BP1 was analyzed as in A. (**C**) HeLa/Flag-Mis18α stable cells were synchronized by double-thymidine block and released into indicated phase. The cells were stained with anti-Flag antibody. The green dots indicate centromeric localization of Mis18α. Confocal image with 1,000x magnification. (**D**) Analysis scheme for the centromere recruitment of newly synthesized CENP-A (left). HeLa cells stably expressing siRNA-resistant form of Mis18α WT (WT^R^) or SA (SA^R^) were transfected sequentially with siRNA against Mis18α and with GFP-CENP-A (mimic newly synthesized CENP-A) as indicated in the scheme. Immunocytochemistry for Mis18α with anti-Flag antibody and GFP-CENP-A (right). (**E**) Scheme for the centromeric recruitment of CENP-A under prolonged Mis18α knockdown (upper). Knockdown of endogenous Mis18α was achieved by infecting lentivirus that is expressing shRNA against Mis18α. Lower left panel shows CENP-A dots in prometaphase cells and lower right panel represents G1 phase cells. (**F**) The number of CENP-A dot positive cells from E were calculated and expressed as a percentage of total cells. *P* value is calculated by *t*-test (^**^p < 0.01).

Since Mis18α plays a role in CENP-A loading process as a licensing or priming factor [4, 7], we questioned whether the phosphorylation of Mis18α by Aurora B kinase is important for this function. Therefore, we first checked the Mis18 complex formation which is essential for CENP-A loading process and found that Mis18α SA has little or no defect in the binding with either Mis18β or M18BP1 (Figure 3A and 3B). Furthermore, Mis18α WT began to show as dots at centromere from anaphase and stayed there until G1 phase (Figure 3C) as reported previously [4]. Mis18α SA showed the similar pattern with WT, indicating that Mis18α phosphorylation at Ser36 by Aurora B kinase is not crucial for its centromere localization.

Next, we examined whether the phosphorylation of Mis18α is required for the CENP-A loading process by adopting and modifying the experimental scheme that was used to show the prerequisite function of Mis18α for the centromere loading of newly synthesized CENP-A [4]. HeLa cells stably expressing siRNA-resistant form of Mis18α WT or Mis18α SA were transfected with siRNA against Mis18α to get rid of endogenous Mis18α and then transfected with GFP-CENP-A as shown in the scheme of Figure 3D. After 24 and 9 hours of siRNA and GFP-CENP-A transfection, respectively, GFP-CENP-A dots were clearly observed at late telophase of both WT and SA-expressing cells (Figure 3D).

We applied an alternative scheme to confirm that Mis18α phosphorylation is unrelated to CENP-A loading process. In this experiment, we could check the effect of prolonged Mis18α knockdown and the reconstitution Mis18α WT or Mis18α SA on CENP-A loading process. HeLa cells were infected with lentivirus expressing shRNA against Mis18α and reconstituted with either Mis18α WT or Mis18α SA as shown in the scheme of Figure 3E. Cells were synchronized by treating with monastrol and then released for 30 min. With the representative data for prometaphase cells (Figure 3E, left) and G1 phase cells (Figure 3E, right), knockdown of Mis18α in HeLa cells diminished CENP-A dots dramatically on day 7; only 13% of cells showed positive signal for CENP-A dots compared with the control shRNA-infected cells (Figure 3F). However, reconstitution of Mis18α WT and Mis18α SA recovered CENP-A dots approximately up to 80% in centromere. Taken together, we could exclude the effect of Mis18α phosphorylation on newly synthesized CENP-A loading into centromere.

### Mis18α phosphorylation enhances PLK1 kinetochore recruitment

**Figure 4:**
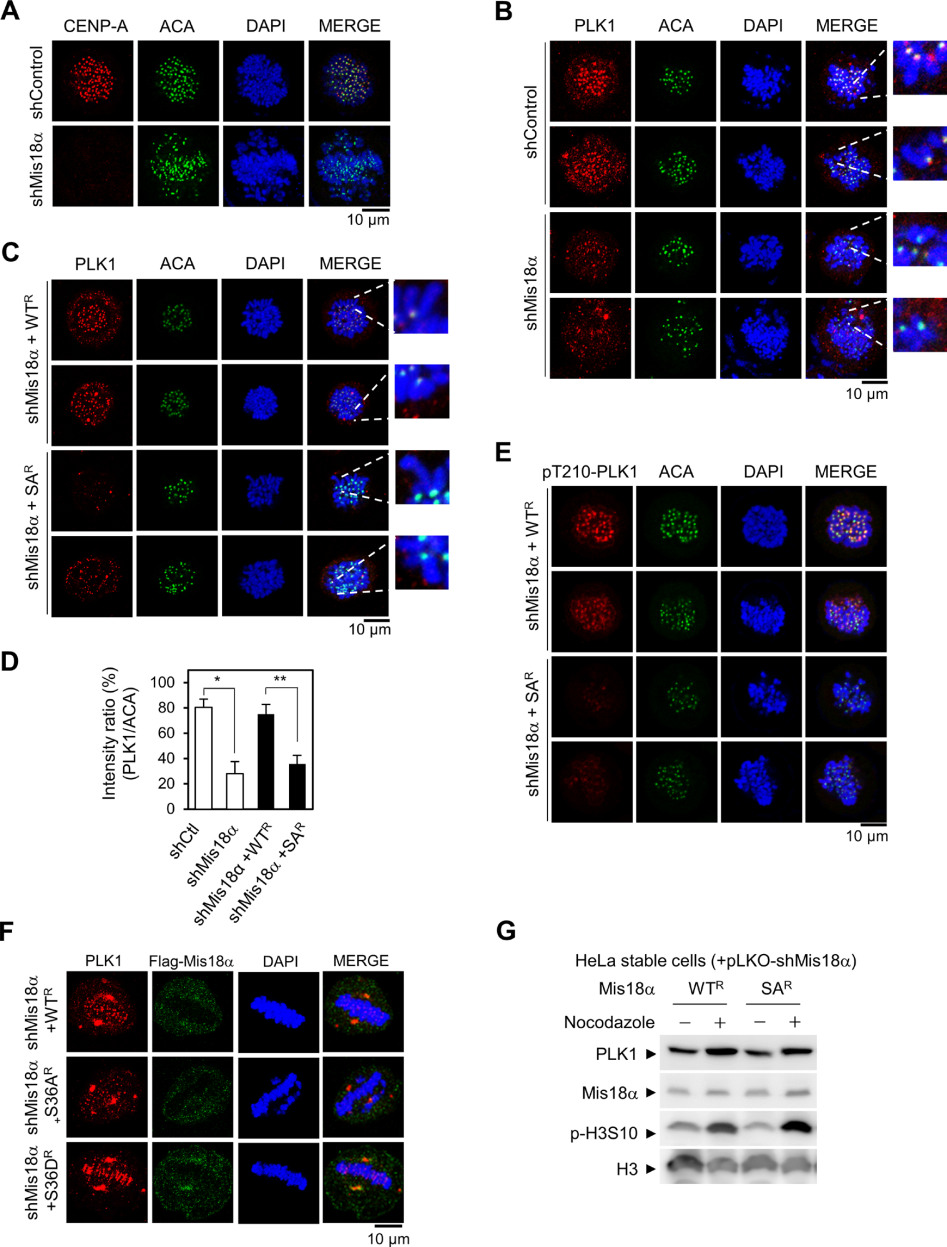
Mis18α phosphorylation enhances PLK1 kinetochore recruitment (**A**) HeLa cells were infected with lentivirus expressing either control shRNA (shControl) or shRNA against Mis18α (shMis18α). Cells were fixed at prometaphase by releasing for 30 min after monastrol treatment and stained with anti-ACA (centromere marker) or anti-CENP-A antibody. Confocal image with 1,000× magnification. (**B**) Cells prepared as in A were co-stained with anti-PLK1 and anti-ACA antibodies. (**C**) HeLa cells stably expressing shRNA-resistant form of Mis18α (WT^R^ and SA^R^) were infected with lentivirus expressing shMis18α. Cells were co-stained with anti-PLK1 and anti-ACA antibodies at prometaphase. (**D**) The number of cells showing high intensity of PLK1 staining, ACA signal as a control (PLK1/ACA), from B and C was presented in percentage. *P* value is calculated by *t*-test (^*^p < 0.05, ^**^p < 0.01). (**E**) pThr210-PLK1 was co-stained with ACA in the same cells as C. (**F**) HeLa cells stably expressing shRNA-resistant form of Mis18α (WT^R^, SA^R^ and SD^R^) were co-stained with anti-PLK1 and anti-Flag Mis18α antibody at metaphase. (**G**) Immunoblot for PLK1 level in reconstituted HeLa stable cell lines.

Aurora B kinase functions to regulate kinetochore-microtubule attachment during prometaphase and PLK1 is another key regulator for this function with Aurora B kinase [14, 20]. To achieve accurate microtubule binding to kinetochore, Aurora B kinase and PLK1 phosphorylate each substrate at a balanced level for microtubule dynamics. If either kinase is abnormally activated, the cells divide with abnormal microtubule binding inducing misaligned chromosomes [14, 20]. We questioned whether the mitotic defects shown in Mis18α SA-reconstituted cells are caused by dysregulation of PLK1 at the kinetochore during prometaphase. Based on this hypothesis, we examined PLK1 recruitment to the kinetochore during prometaphase compared to *Mis18α*-depleted HeLa cells. Mis18α knockdown was validated by the disappearance of CENP-A dots, while anti-centromere antibody (ACA) recognized centromere regions in the chromosomes (Figure 4A). In control cells, PLK1 dots were clearly stained consistently with centromere markers, whereas the intensity of PLK1 dots was significantly reduced at the kinetochore in *Mis18α*-knockdown cells (Figure 4B), indicating that Mis18α is necessary for the proper recruitment of PLK1 to the kinetochore. Interestingly, introduction of Mis18α WT recovered the intensity of PLK1 dots; however, introduction of Mis18α SA was not sufficient to substitute for Mis18α WT (Figure 4C). The evaluation of the ratio of PLK1 dots to ACA centromere maker confirmed that Mis18α phosphorylation is necessary for the proper recruitment of PLK1 to kinetochore (Figure 4D). Since Aurora B is also responsible for PLK1 activation at kinetochore through Thr210 phosphorylation [14], we next checked whether Mis18α phosphorylation is also involved in it. Interestingly, Thr210 phosphorylation of PLK1 was also decreased in Mis18α SA-reconstituted cells, indicating that Mis18α phosphorylation is essential for the function of PLK1 at kinetochore (Figure 4E). Moreover, Mis18α SD, a phospho-mimic form, recovered and further maintained PLK1 recruitment even during the metaphase when PLK1 starts to leave kinetochore [20] (Figure 4F). The level of PLK1 in Mis18α WT- or Mis18α SA-reconstituted cells was comparable, indicating that reduced kinetochore recruitment of PLK1 in Mis18α SA-reconstituted cells is not related to its protein level (Figure 4G). Taken together, these results indicate that Mis18α phosphorylation by Aurora B kinase is necessary for the PLK1 kinetochore localization at early mitosis.

### PLK1 recognizes Mis18α phosphorylation through its Polo Box Domain (PBD)

**Figure 5:**
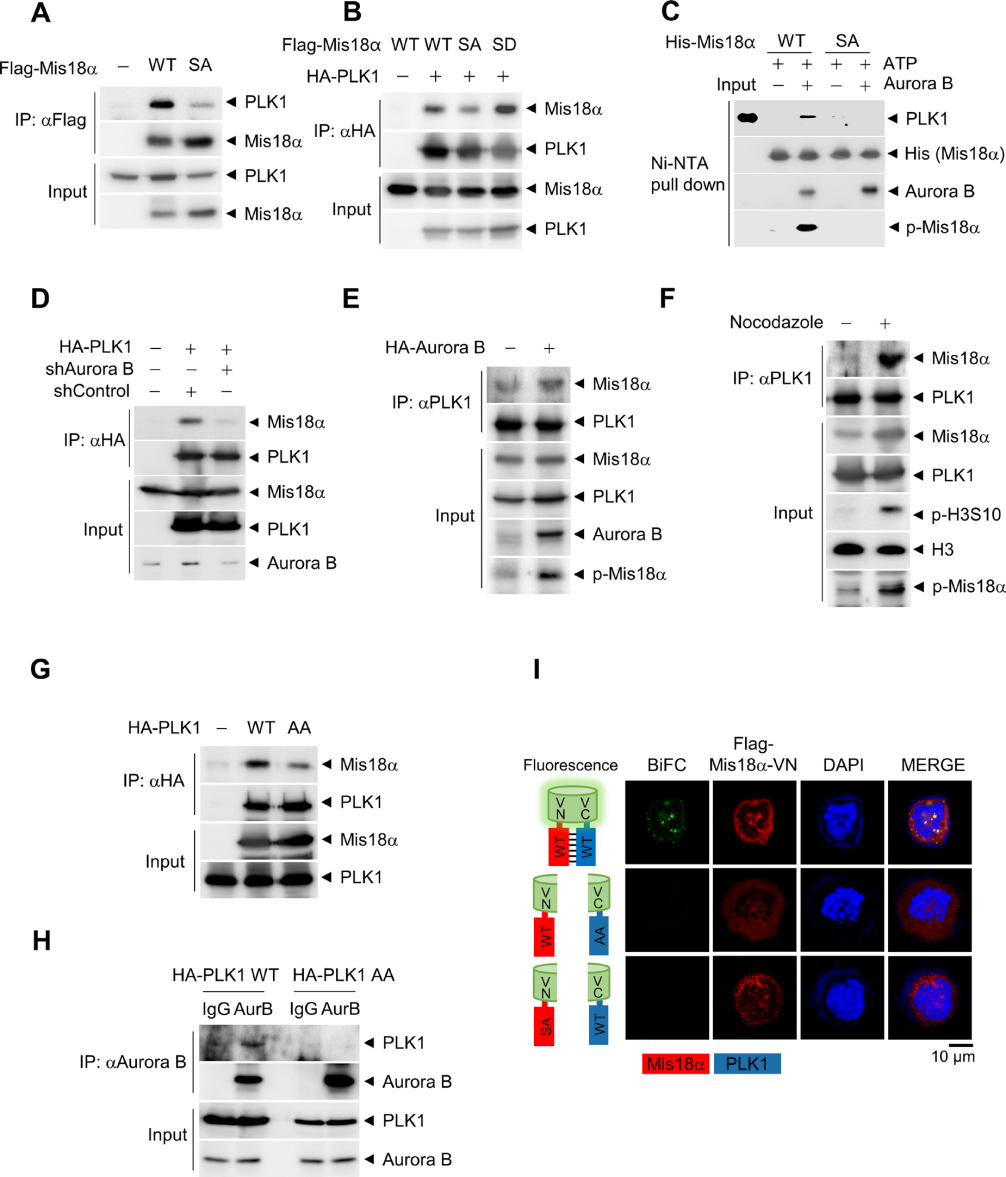
PLK1 recognizes Mis18α phosphorylation through its PBD (**A**) Flag-Mis18α and HA-PLK1 constructs were transfected in 293T cells and cell extracts were applied for IP analysis by using anti-Flag antibody. (**B**) Flag-Mis18α and HA-PLK1 constructs were transfected in 293T cells and cell extracts were applied for IP analysis by using anti-HA antibody. (**C**) HA-PLK1 was synthesized *in vitro* by using a coupled Transcription/Translation system and incubated with recombinant His-Mis18α in the presence of Aurora B kinase for *in vitro* binding assay. The sample was subjected to immunoblotting with anti-HA antibody. (**D**) 293T cells were transfected as in A in the presence or absence of shRNA against Aurora B kinase. IP was performed by using anti-HA antibody. (**E**–**F**) Flag-Mis18α constructs were transfected in 293T cells in the presence of Aurora B kinase overexpression (E) or nocodazole treatment (F), and IP was performed by using anti-PLK1 antibody. (**G**) Flag-Mis18α was transfected into 293T cells together with either wild-type PLK1 (HA-PLK1 WT) or PBD-mutant form of PLK1 (HA-PLK1 AA). IP was performed using anti-HA antibody. (**H**) 293T cell extracts expressing either HA-PLK1 WT or HA-PLK1 AA were applied for IP analysis using anti-Aurora B kinase antibody. (**I**) Bimolecular fluorescence complementation assay. Flag-Mis18α-VN constructs and HA-PLK1-VC constructs were transfected into HeLa cells and the fluorescence images were detected under confocal microscope (green fluorescence for BiFC and red fluorescence for Mis18α). Confocal image with 1,000× magnification.

To examine whether Mis18α-PLK1 binding is important for PLK1 kinetochore recruitment and whether Mis18α phosphorylation mediates the binding, we performed co-immunoprecipitation assay. Interestingly, PLK1 bound with Mis18α WT, but the binding with Mis18α SA decreased significantly (Figure 5A). In addition, the binding between PLK1 and Mis18α SD phospho-mimic mutant increased more than Mis18α WT (Figure 5B). *In vitro* binding assay was also performed to confirm if Mis18α phosphorylation is necessary for its interaction with PLK1. Mis18α interacted with PLK1 only in the presence of ATP and Aurora B kinase, whereas Mis18α SA did not bind with PLK1 even in the presence of ATP and Aurora B kinase (Figure 5C). In a separate assay, phosphorylation mimic mutant form of Mis18α (Mis18α SD) interacted with PLK1 without addition of Aurora B kinase (Supplementary Figure 5). Furthermore, knockdown of Aurora B kinase diminished the binding between PLK1 and Mis18α (Figure 5D), and overexpression of Aurora B kinase increased their binding (Figure 5E), revealing Aurora B kinase dependent binding of PLK1. Nocodazole treatment increased the binding between PLK1 and Mis18α (Figure 5F). Thus, phosphorylation of Mis18α enhances its binding with PLK1.

**Figure 6:**
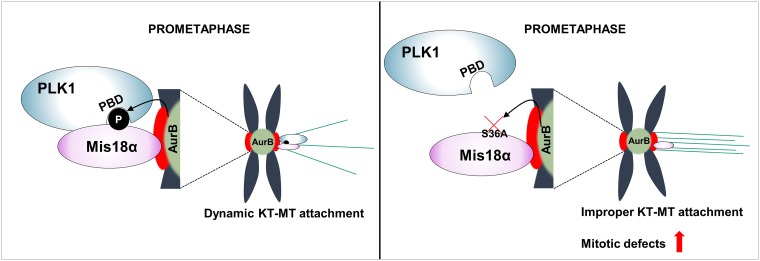
Mis18α function during mitosis by enhancing PLK1 kinetochore recruitment A schematic model showing how Mis18α phosphorylation enhances PLK1 recruitment at the kinetochore.

PLK1 localization is enhanced by phospho-binding domain, called Polo Box Domain (PBD) [21, 22]. PLK1 recognizes substrates through its PBD, and substrate binding through PBD leads to further activation of PLK1. Therefore, we checked whether the PBD of PLK1 is involved in the binding with the phosphorylated Mis18α. Since two sites (His538 and Lys540) in PBD are important for the binding of PLK1 to other substrates [23], we generated PLK1 AA mutant by substituting each amino acid to alanine. Interestingly, the binding of PLK1 AA with Mis18α decreased considerably compared to PLK1 WT (Figure 5G). PLK1 AA mutant also exhibited decreased binding to Aurora B kinase (Figure 5H). To gain more insight into PLK1 and Mis18α interaction, we performed bimolecular fluorescence complementation assay [24]. The N-terminal half and the C-terminal half of VENUS were fused with Mis18α and PLK1, respectively. When the two proteins were co-expressed, Mis18α/PLK1 interaction-mediated complementation of VENUS showed green fluorescence signals. However, Mis18α SA or PLK1 AA mutant failed to show any fluorescence signal (Figure 5I). Taken together, PLK1 binds to phosphorylated Mis18α through its PBD and the binding enhances PLK1 recruitment to the kinetochore at prometaphase (Figure 6).

## DISCUSSION

Mis18α is a component of Mis18 complex that is crucial for centromere deposition of newly synthesized CENP-A at early G1 phase of cell cycle [4, 6]. In this study, we identified a distinct role of Mis18α at mitosis, independent of its known function in CENP-A deposition at early G1 phase. The mitotic function of Mis18α is accompanied by Aurora B kinase-mediated phosphorylation on a conserved serine residue (Ser36 in human and Ser13 in mouse) during mitosis and this phosphorylation enhances the interaction between Mis18α and PLK1 resulting in the increased kinetochore recruitment of PLK1. The Mis18α phosphorylation we have shown here is distinct from previously identified phosphorylations of Mis18 proteins by CDK1/2 and PLK1, which mainly regulate the function for CENP-A deposition. First, it occurs during mitosis specifically by Aurora B kinase, and the association with Mis18BP1 is not necessary for the phosphorylation. Second and most importantly, the phosphorylation is independent of CENP-A loading. Phosphorylation-defective mutation of Mis18α does not affect either timely scheduled centromere localization of Mis18 complex during cell cycle progression or its function as a priming factor for CENP-A deposition. In detail, the replacement of endogenous Mis18α with phosphorylation-defective Mis18α in two cell types, HeLa and MEFs, did not induce CENP-A loss, whereas Mis18α depletion without Mis18α reconstitution clearly caused CENP-A loss.

It has been previously shown that the depletion of Mis18α causes mitotic defects such as misaligned chromosomes and chromosomal bridges [3], which finally leads to cell death resulting in developmental failure of embryos and skin stratification in mice [3, 5]. Interestingly, the expression of phosphorylation-defective mutant form of Mis18α in *Mis18α*-depleted cells can rescue severe lethal phenotype of *Mis18α* depletion; however, still exhibits mitotic defects, indicating that although the phosphorylation is not critical for CENP-A deposition, it contributes to the faithful chromosome alignment and segregation. Indeed, Aurora B kinase and PLK1 are essential kinases for accurate KT-MT attachment by interdependent regulation and the loss of Aurora B kinase activity is known to cause abnormally stable KT-MT attachment, as the cooperation with PLK1 to balance KT and MT tension is collapsed [25–28]. This results in abnormally increased inter-kinetochore distance [29]. Aurora B kinase destabilizes KT-MT attachment, whereas PLK1 stabilizes it by phosphorylating BubR1 to disturb Aurora B kinase activity [25]. However, PLK1 activates Aurora B kinase by phosphorylating Survivin, which is a member of chromosomal passenger complex and Aurora B kinase activates PLK1 by direct phosphorylation [14, 15]. Due to this complicated cooperation, cells may have multilayers of self-checking system for accurate KT-MT attachment and Mis18α phosphorylation by Aurora B kinase would have a role of enhancing PLK1 recruitment.

The most striking feature of PLK1 regulation is PBD-dependent interaction with its substrates resulting in the kinetochore localization. There are many studies on the proteins that recruit PLK1 to the kinetochore by investigating their interaction with PBD. The PBIP1 recruits PLK1 by PBD-mediated binding at early mitosis and PLK1 phosphorylates PBIP1 for self-primed enrichment [30]. Interestingly, PLK1-mediated phosphorylation of PBIP1 on another site induces its degradation and releases PLK1 for the interaction with other recruiting factors. Indeed, PLK1 kinetochore signals remain even when the PBIP1 is depleted [31], indicating multilayered regulation of PLK1 kinetochore recruitment. In addition, INCENP and RSF1 are known to function in PLK1 kinetochore recruitment. Knockdown of INCENP, which is a member of passenger complex with Aurora B kinase, resulted in the loss of PLK1 kinetochore recruitment [32] providing the evidence of Aurora B complex's involvement in PLK1 recruitment. RSF1 partially regulates PLK1 kinetochore recruitment and dual knockdown of RSF1 and INCENP further reduces PLK1 kinetochore recruitment [33]. In our study, Mis18α SA reconstitution did not completely inhibit PLK1 recruitment to the kinetochore. Moreover, there was no obvious mitotic delay. Therefore, we speculate that the mitotic defects observed from Mis18α SA-reconstituted cells would be the accumulated defects from prolonged dysregulation of PLK1.

In summary, we have identified mitosis-specific phosphorylation of Mis18α by Aurora B kinase, which is not essential for the previously known function of Mis18 complex in CENP-A deposition to centromere. Instead, the phosphorylation of Mis18α contributes the recruitment of PLK1 to the kinetochore, which requires PBD-mediated binding of phosphorylated Mis18α at prometaphase. Since the phosphorylation-defective mutation on Mis18α causes mitotic problems, Mis18α plays a critical role in mitosis in addition to its well-known function in CENP-A deposition at G1 phase. Our finding opens up a possibility that Mis18α may play a diverse role in a wide range of cell cycle regulation.

## MATERIALS AND METHODS

### Cell culture, generation of stable cells and transfection

HeLa, 293T and *Mis18α^f/f^/ESR-Cre* MEFs were cultured in 37°C humidified CO_2_ incubator with DMEM containing 10% FBS and antibiotics. All cell lines were regularly tested for mycoplasma contamination. For the generation of Mis18α-stably reconstituted HeLa cell lines, cells transfected with shRNA-resistant Flag-Mis18α were selected with neomycin for two weeks. The cells were then infected with shMis18α expressing lentivirus (pLKO-shMis18α) followed by selection with puromycin. Lentivirus was generated by transfecting lentiviral shRNA and packaging plasmids (psPAX2 and pMD2.G) into 293T cells. The culture supernatant were collected two days later and concentrated by Lenti-concentrator (Takara Bio, USA). The targeting sequences of shRNA are as follows; human Mis18α, 5′-CAGAAGCTATCCAAACGTG-3′; human M18BP1, 5′-GGATATCCAAATTATCTCA-3′. The targeting sequence of siRNA for human Mis18α is as follows; 5′-CAGAAGCUAUCCAAACGUGUU-3′. For the generation of Mis18α-reconstituted *Mis18α^f/f^/ESR-Cre* MEF cell lines, cells were infected with Flag-mMis18α expressing lentivirus (pLJM1-Flag-Mis18α) and selected with puromycin for two weeks. For the depletion of endogenous mMis18α, 4-hydroxy-tamoxifen (200 nM) was added for 4 days.

### Cell synchronization

To arrest cells at G1/S, the cells were incubated with DMEM media containing 4 mM thymidine (Sigma, St Louis, MO) for 16 h and were washed with PBS twice. After 9 h of release, cells were incubated with thymidine containing media again for 15 h, and were released and harvested at indicated time points. To arrest cells at metaphase, the cells were incubated with DMEM media containing 0.4 μg nocodazole (Sigma, St Louis, MO) for 15 h. For metaphase-aligned chromosomes, MG132 (Sigma, St Louis, MO) was added while cells were released from 100 nM monastrol incubation for 15 h.

### Bimolecular fluorescence complementation assay

The N-terminal half of Venus fluorescent protein with Flag-tag was fused with Mis18α and the C-terminal half of Venus with HA-tag was fused with PLK1. Phosphorylation-defective mutant of Mis18α and PBD mutant of PLK1 were fused with Venus in the same way, respectively. These constructs were transfected into HeLa cells in combination. The cells were synchronized by monastrol treatment and then released to obtain prometaphase population. The expression of each construct was validated by immunostaining with anti-Flag or anti-HA antibodies. The complemented Venus protein was detected by green fluorescence signal using a confocal microscope (Carl Zeiss, Germany).

### Immunoprecipitation

The whole cell lysates were prepared using lysis buffer (50 mM Tris-HCl pH 8.0 containing 200 mM NaCl, 0.5% NP-40, and freshly added protease and phosphatase inhibitors). Cell lysates were briefly sonicated to shear the chromatin structure. For immunoprecipitation, 1 mg of lysates were incubated sequentially with primary antibody for 4 h followed by protein A/G coated beads for 1 h at 4°C.

### Immunoblot

For immunoblot, normalized cell lysates or immunoprecipitation samples were separated on SDS-PAGE gels and transferred on nitrocellulose membrane (GE Healthcare, Little Chalfont, UK). The blots were probed with the following primary antibodies; anti-Flag (Sigma, St Louis, MO), anti-HA (Covance, Princeton, NJ), anti-H3 (Cell signaling, Danvers, MA), anti-p-H3S10 (Abcam, Cambridge, UK), anti-Aurora B (Abcam, Cambridge, UK), anti-PLK1 (Santa Cruz, Dallas, TX), anti-ACA (Antibodies Incorporated, Davis, CA), anti-CENP-A (Cell Signaling, Danvers, MA), anti-phospho serine (Sigma, St Louis, MO). Phosphorylation-specific antibody for Mis18α was generated by injecting synthetic phospho-peptide to rabbits and purified using phospho-peptide affinity chromatography (AbClone, Seoul, South Korea). Peptide sequence used for injection is as follows; 5′-CESPLLEKRL(pS)EDSSR-3′.

### Immunocytochemistry

For immunocytochemistry, the cells were cultured on poly-L-Lysine coated coverslip or chamber slide and were fixed with 2% formaldehyde for 15 min at 25°C. Cells were then permeabilized with PBS containing 0.5% Triton X-100 for 5 min followed by incubation with primary antibodies for overnight. After washing, secondary antibodies conjugated with Alexa Fluor 488 or 594 (Invitrogen, Carlsbad, CA) were applied in the dark for 1 h at 25°C. DAPI was incubated for short time just before mounting and the slides were observed with confocal microscope (Carl Zeiss, Germany) at 1,000× magnification with immersion oil. The images were analyzed by Image J software.

### *In vitro* binding assay

HA-PLK1 was synthesized *in vitro* by using a coupled transcription and translation systems (Promega, USA). HA-PLK1 was incubated with recombinant GST-Mis18α for GST-pull down in binding buffer (125 mM NaCl, 20 mM Tris-HCl pH 8.0, 10% Glycerol, 0.1% NP-40, 0.5 mM DTT and protease inhibitors). The reaction was stopped by adding SDS sampling buffer and was subjected to SDS-PAGE. PLK1 was analyzed by immunoblotting with anti-HA antibody. Amount of GST-Mis18α was analyzed by anti-GST antibody.

### *In vitro* kinase assay

Recombinant His-H3 and His-Mis18α was incubated with purified Aurora B kinase (Eurofins, UK) for 30 min at 30°C in the kinase buffer (50 mM Tris-HCl, 10 mM MgCl_2_, 1 mM EGTA, 2 mM DTT, 0.01% Tween-20, 1 mM ATP, protease inhibitors and phosphatase inhibitors). The reaction was stopped by adding SDS sampling buffer and subjected to SDS-PAGE followed by immunoblotting with anti-p-H3S10 antibody and anti-p-Mis18α antibody.

### *In vitro* kinase-binding assay

Recombinant His-Mis18α that is bound on Ni-NTA-agarose was incubated with purified Aurora B kinase (Eurofins, UK) for 30 min at 30°C in the kinase buffer. The sample was washed with binding buffer and then incubated with *in vitro* synthesized HA-PLK1 in binding buffer for 2 h with rotating. After collecting the beads and washing with binding buffer, samples were boiled for 5 min with SDS sampling buffer and subjected to SDS-PAGE. PLK1 was analyzed by immunoblotting with anti-HA antibody.

### Chromosome spreading assay

*Mis18α^f/f^/ESR-Cre* WT MEFs and *Mis18α^f/f^/ESR-Cre* SA MEFs were incubated with colcemid (Sigma, USA) to a final concentration of 1 μg/ml for 4 h at 37°C. After incubation, cells were trypsinized and harvested by centrifugation at 1,000 rpm for 4 min. The pellet was resuspended with 75 mM KCl solution and incubated for 6 min. After harvesting by centrifugation at 1,000 rpm for 4 min, cells were fixed with methanol/glacial acetic acid (3:1) by dropping and mixing slowly. The fixed chromosomes were released as a single drop at a time onto the slide and were allowed to air-dry. The air-dried slide was covered by coverslip with DAPI-containing mounting solution. The image was observed by confocal microscope (Carl Zeiss, Germany) at 1,000x magnification, and centromeres that are brighter than the rest of the chromosome were counted by Image J software.

### Statistical analysis

All experiments were performed independently at least three times. More than 100 cells were counted to evaluate mitotic defects in each experiment. For PLK1/ACA intensity ratio, an average number of 150 kinetochores was examined for each group. Values are expressed as mean ± s.e.m. Significance was analyzed using two-tailed, unpaired *t*-test. *P* < 0.05 was considered statistically significant.

## SUPPLEMENTARY MATERIALS FIGURES



## Abbreviations

MT: Microtubule
KT: Kinetochore
SAC: Spindle Assembly Checkpoint
PBD: Polo Box Domain
M: Mitosis
