# Supplementary material for: Mitosis-specific phosphorylation of Mis18α by Aurora B kinase enhances kinetochore recruitment of polo-like kinase 1
Source: Oncotarget. 2017 Nov 27;9(2):1563–76. doi: 10.18632/oncotarget.22707 (PMC5788582; doi:10.18632/oncotarget.22707)
Supplement: Supplementary file 1 [file oncotarget-09-1563-s001.pdf]

Mitosis-specific phosphorylation of Mis18α by Aurora B kinase enhances kinetochore recruitment of polo-like kinase 1

SUPPLEMENTARY MATERIALS

LC-MS/MS analysis of Mis18α phosphorylation in mitosis

Flag-Mis18α was overexpressed in 293T cells and the cells were treated with nocodazole for 15 h. The lysates were subjected to overnight incubation with anti-Flag M2 beads. The beads were washed with lysis buffer and eluted with 3xFlag peptide. The eluent was subjected to SDS-PAGE. The gel slice corresponding to Flag-Mis18α was destained in 50% ACN solution of 25 mM NH_4HCO_3 buffer for 10 min and then followed by in-gel alkylation of cysteine residues with dithiothreitol and iodoacetamide. After three times washing out the excess alkylation reagents with 25 mM NH_4HCO_3, the resulting sample was digested by sequencing-grade trypsin at ratio of 1:50 (w/w) for overnight at 37°C. The digested peptides were subjected to C18-SPE clean up using 10 µL of ZipTip (Millipore). The final peptides sample was reconstituted with 25 mM NH_4HCO_3 for LC-MS/MS analysis. LC-MS/MS experiment with HCD fragmentation mode was performed on Orbitrap Fusion Lumos mass spectrometry (Thermo Fisher Scientific) coupled with nanoACQUITY UPLC (Waters) equipped with an in-house packed capillary trap column (150 µm i.d. × 3 cm) and analytical column (75 µm i.d. × 100 cm) with 3 µm Jupiter C18 particles (Phenomenex). The acquired dataset was searched by MS-GF+ algorithm at 10 ppm of precursor ion mass tolerance against the SwissProt Homo sapiens proteome database.

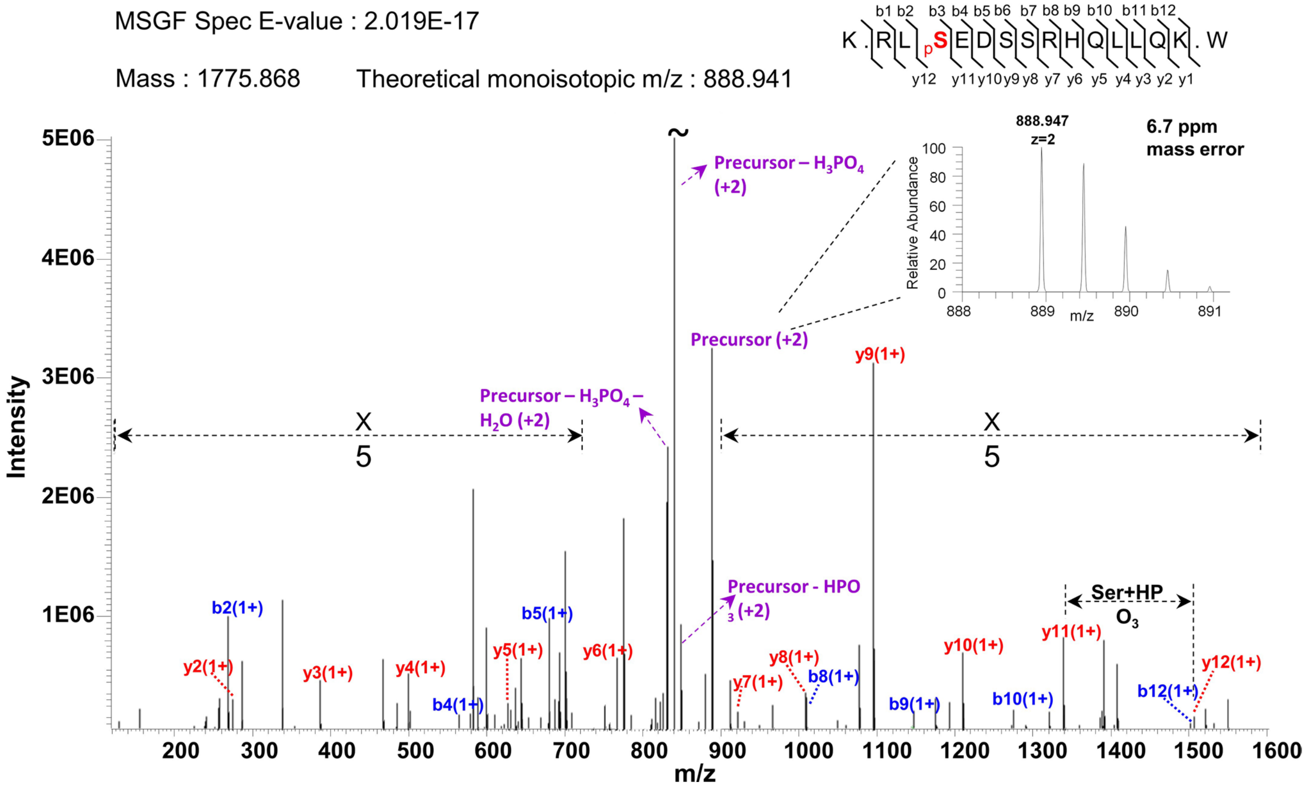

Supplementary Figure 1: LC-MS/MS analysis of Mis18α phosphorylation in mitosis. Flag-Mis18α was overexpressed in 293T cells and the cells were treated with nocodazole for 15 h. The lysates were subjected to overnight incubation with anti-Flag M2 beads. The beads were washed with lysis buffer and eluted with 3xFlag peptide. The elute was subjected to SDS-PAGE and the gel slice corresponding to Flag-Misl8α was used for subsequent LC-MS/MS analysis.

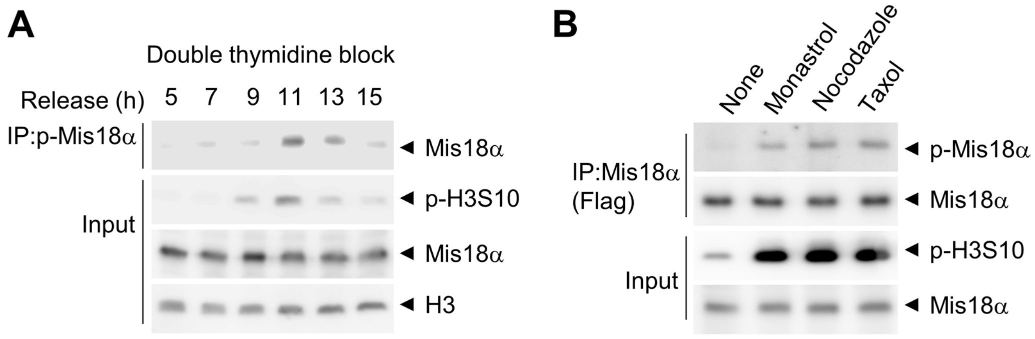

Supplementary Figure 2: Analysis of the time of Mis18α phosphorylation during cell cycle using phospho-Ser36 specific antibody. (**A**) HeLa cells stably expressing Flag-Mis18α (HeLa/Flag-Mis18α) were synchronized at G liS by double thymidine block and released into indicated time points. Cell lysates were subjected to immunoprecipitation with anti-p-Mis18α antibody and analyzed by immunoblotting with anti-Flag (Mis18α) antibody. (**B**) HeLa/FlagMis 18a cells were synchronized by monastrol, nocodazole, or taxol and the cells were collected by shake-off. Cell lysates were subjected to immunoprecipitation with anti-Flag (Mis18α) antibody and analyzed by immunoblotting with anti-p-Mis18α antibody.

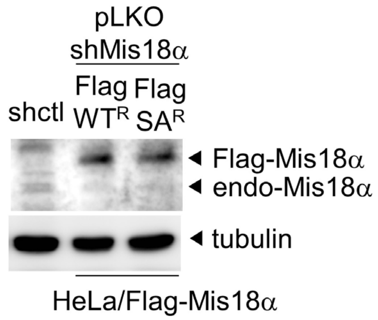

Supplementary Figure 3: Knockdown efficiency of endogenous Mis18α. HeLa cells stably expressing shRNA resistant form of Flag-Mis18α (WTR or SAR) were infected with lentivirus generated from pLKO-shMis18α. Regular HeLa cells infected with lentivirus generated from shRNA pLKO-shControl (shctl) were used as a control. The knockdown efficiency was evaluated by immunoblot analysis with anti-Mis18α antibody.

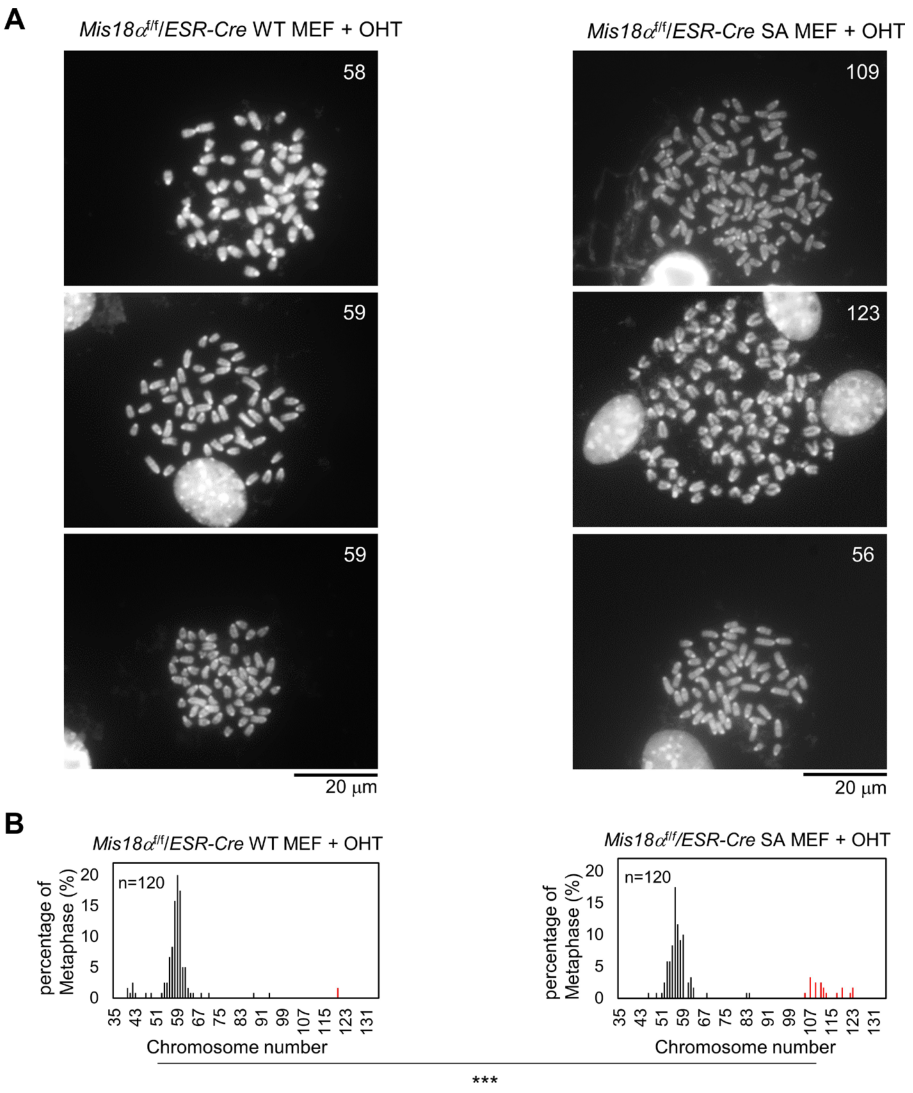

Supplementary Figure 4: Chromosome spreading assay. (**A**) Mis18c11flESR-Cre WT MEFs and Mis18c11flESR-Cre SA MEFs were treated with 4-0HT for 4 days. The number of mitotic chromosomes were examined by chromosome spreading assay from each MEFs. Photographs are representative pictures for each MEFs. Confocal images with 1,000× magnification. (**B**) Histogram shows the percentage of cells with respective numbers of chromosomes. The number of chromosome spreads is total 120 (three times of experiment with 40 spreads each). Chromosomes were counted by identifying the centromeres, the brighter part than the rest of the chromosome, using Image J software. *P* value is calculated by *t*-test ($^{***}p < 0.001$).

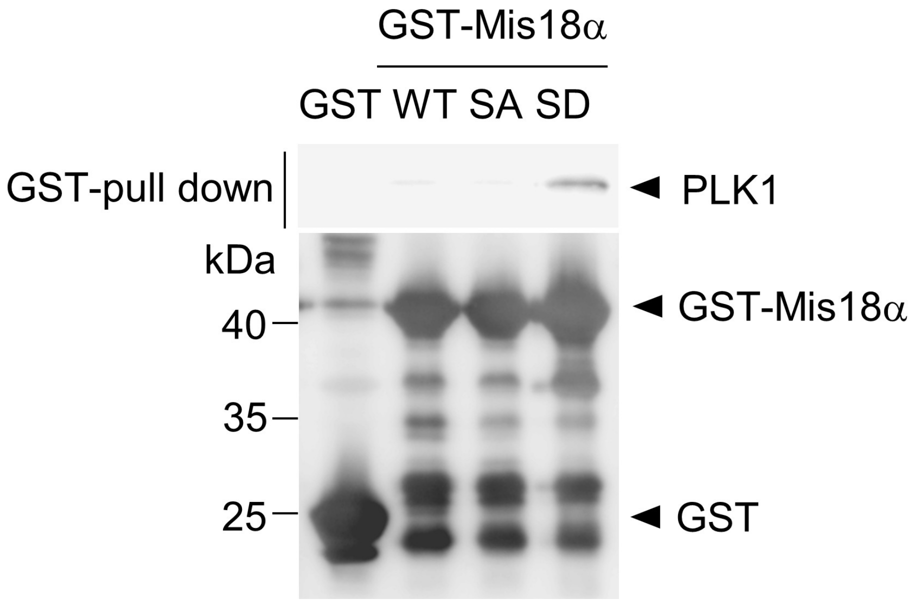

Supplementary Figure 5: *In vitro* binding assay between recombinant Mis18α and PLKI. HA-PLKI was synthesized *in vitro* by using a coupled Transcription/Translation system and incubated with bacterially expressed recombinant GST-Mis18α. After GST-pull-down, samples were separated on SDS-PAGE and analyzed by immunoblotting with anti-HA antibody.
